# Improvement of a coastal vulnerability index and its application along the Calabria Coastline, Italy

**DOI:** 10.1038/s41598-022-26374-w

**Published:** 2022-12-19

**Authors:** Daniela Pantusa, Felice D’Alessandro, Ferdinando Frega, Antonio Francone, Giuseppe Roberto Tomasicchio

**Affiliations:** 1 Faculty of Engineering, eCampus University, 22060 Novedrate, CO Italy; 2 Department of Environmental Science and Policy, University of Milan, 20122 Milan, Italy; 3 Department of Civil Engineering, University of Calabria, 87036 Arcavacata di Rende, CS Italy; 4 Department of Engineering for Innovation, University of Salento, 73100 Lecce, Italy

**Keywords:** Environmental sciences, Natural hazards

## Abstract

The present paper further develops a coastal vulnerability index formulation (CVI) previously proposed by the authors by integrating a new variable and redefining three variables to improve the suitability of the index for low-lying coasts. Eleven variables are divided into three typological groups: geological, hydro-physical process and vegetation. The geological variables are: geomorphology, shoreline erosion/accretion rates, coastal slope, emerged beach width, and dune. The hydro-physical process group includes: river discharge, sea-level change, mean significant wave height and mean tide range. The vegetation variables are: vegetation behind the back-beach and coverage of *Posidonia oceanica*. The index was applied to a stretch of the Ionian coast in the province of Crotone in the Calabria region (Southern Italy), and a vulnerability map was produced. A geography information system (GIS) platform was used to better process the data. For the case study area, the most influential variables are shoreline erosion/accretion rates, coastal slope, emerged beach width, dune, vegetation behind the back-beach, and coverage of *Posidonia oceanica*. The most vulnerable transects are those near urban areas characterized by the absence of dunes and vegetation. Statistical and sensitivity analyses were performed, and the proposed CVI was compared with the previous formulation proposed by the authors and with two other CVI methods present in the literature.

## Introduction

Coastal areas are dynamic environmental and socioeconomic systems that provide services including wildlife habitat, erosion and flooding protection, and economic and recreational activities. These areas are vulnerable to a number of factors, including erosion, subsidence and changes in hydrology, and are often characterized by increasing population densities^1^.

Defining simple predictive approaches is essential for assessing and categorizing responses to progressive changes in coastal dynamics around the world.

Gornitz, White and Cushnam^2^ and Gornitz^3^ originally proposed a coastal vulnerability index (CVI), as an index-based method to assess coastal vulnerability to climate change, particularly to sea level rise (SLR). Thieler and Hammar-Klose^4^ proposed a new CVI formulation that modified the original index developed by^2,3^, resulting in a widely used tool for applications at different territorial scales. Furthermore, several CVI formulations have since been proposed with modifications and integrations of the original physical parameters to adapt the index to the particular characteristics of the coastal area.

Specifically, applications have been carried out for different coastal areas worldwide, such as the U.S. coast^5–7^, Indian coast^8–11^, Australian coast^12,13^, African coast^14–17^, and European coast^18–20^. Formulations combining physical and socioeconomic variables have also been developed^21–26^.

In this context, the present paper resumes and continues a previous CVI formulation developed by Pantusa et al.^27^ and proposes improvements particularly suitable for low-lying coasts. The interest in low-lying coasts is motivated by the desire to (i) identify their high and growing exposure to different hazards, such as, storm surges, flooding, and sea level rise^28,29^, and, consequently, (ii) design coastal interventions^30–34^ and (iii) orient planning strategies. The improved CVI index has been applied to a stretch of the Mediterranean low-lying coast , in the Calabria region, Italy, an area impacted by intense human activity and naturally exposed to climate and environmental changes that determine coastal erosion, extreme events, sedimentation decrease and degradation of some habitats (e.g. coastal dunes, coastal cliffs or coastal terraces)^35–38^.

With specific reference to low-lying coasts, several CVI formulations have been proposed in the literature. Some studies refer to the variables and formulation originally proposed by^2–4^, while others include additional parameters.

Several studies are summarized in Table 1, with specific reference to the adopted method, geographical location, variables and remarks.

**Table 1:** CVI formulations applied to low-lying coasts.

Method	Reference	Geographical location	Variables	Remarks
Coastal vulnerability index (CVI)	Dwarakish et al.^8^	India	Geomorphology, shoreline change, coastal slope, mean tidal range, mean significant wave height, mean sea level rise	6 Geological and physical variables
Coastal vulnerability index (CVI)	Appeaning Addo^14^	Ghana	Geology, geomorphology, elevation, relative sea-level rise rates, shoreline recession rates, tide range, mean wave height	7 Geological and physical variables
Improved coastal vulnerability index (ICVI)	El-Hattab^16^	Nile Delta	Geology, elevation, coastal slope, geomorphology, shoreline change, presence of coastal protection, relative sea level rise, land subsidence, population density, socio-economic status	10 Geologic, physical and socio-economic variables
Coastal vulnerability index (CVI)	Sekovsky et al.^20^	Italy	Elevation, dunes, artificial protection structures, shoreline change rates, land cover	5 Physical variables
Coastal vulnerability index (CVI)	Mani Murali et al.^24^	India	Slope, geomorphology, elevation, shoreline change, sea level rise, significant wave height tidal range, population, land use/land cover, roads and location of tourist areas	7 Physical and 4 socio-economic variables; combination of physical vulnerability index (PVI) and socio-economic vulnerability index (SVI)
Coastal vulnerability index (CVI)	Bagdanavičiūtė et al.^39^	Lituania	Shoreline change, beach width/height, underwater slope, sand bars, and beach sediments, significant wave heigh	5 Geologic and physical variables
Coastal vulnerability index (CVI)	Pramanik et al.^40^	Mumbai	Shoreline change, coastal elevation, coastal slope, geomorphology, bathymetry, sea level rise, significant wave eight, mean tidal range, LU/LC, population density, tourist density, fisherfolk density	5 Geological, 3 physical and 4 socio-economic variables
Coastal vulnerability index (CVI)	Gaki-Papanastassiou et al.^41^	Greece	Geomorphology, coastal slope, rate of relative, sea-level rise, ate of shoreline erosion/accretion, mean tide range, mean significant wave height	6 Geologic and physical variables
Coastal sensitive index (cSI)	Karymbalis et al.^42^	Greece	Geomorphology, coastal slope, rate of relative sea-level rise, rate of shoreline erosion/accretion, mean tide range, mean significant wave height	6 Geologic and physical variables
CVI and SVI	Tragaki et al.^43^	Greece	Geomorphology, shoreline change, coastal slope, relative sea level rise, significant wave height, tidal range; population density, share of women in total population, share of persons above 65 in total population, share of children below 5 in total population, share of foreign-born in total population, share of low educated in total population	6 Geologic and physical variables and 7 social variables; coastal vulnerability index (CVI) and social vulnerability Index (SVI)

As described in Table 1, some applications to low-lying coasts^8,41,42^ consider only the variables originally proposed by^4^, while the index proposed by^24^ adds a further geological variable. These applications focus on physical vulnerability and do not integrate further specific variables for low-lying coasts.

Other indices^16,24,40,43^ extend the original CVI formulation, also taking into account socioeconomic factors. Therefore, these indices, which combine physical and socioeconomic variables, assess coastal vulnerability by a different approach compared to that proposed in this work.

In the approach proposed by Bagdanavičiūtė et al.^39^, the index relies almost exclusively on geological variables and uses also some variables suitable for low-lying coasts. Similarly, the index proposed by Sekosky et al.^20^ includes some variables specific for low-lying coasts.

Compared to these indices, the novelty of the proposed index is the combination of all the variables originally proposed by^4^ with further specific variables that take into account the recognized factors of interest for low-lying coasts from climate-related changes: dunes, vegetation, and marine ecosystems^44^. The addition and combination of these variables with the original CVI geological and physical variables allows us to undertake a more specific analysis of the coastal response to sea natural hazards and coastal inundation that constitute threats, especially for low-lying coasts. A more in-depth analysis, especially at the local scale, can be useful to better identify the most vulnerable transects and can better support policy and management tools, especially when investment budgets are limited.

As mentioned above, the present work further develops a CVI index previously proposed by the authors that integrates a new variables and redefines three variables. The variables are divided into three typological groups: geologic, hydro-physical process and vegetation.

Statistical analysis was carried out to evaluate the correlation and multicollinearity between the considered variables, together with a sensitivity analysis of the variable ranking score, aggregation formulation and overall CVI classification method. Considering the three typological groups of variables, statistical analysis was also performed to identify the groups that predominantly influenced the overall coastal vulnerability.

Finally, an application of the CVI formulation previously proposed by the authors^27^ and of two other CVI methods proposed in the literature by Ružić et al.^45^ and Palmer et al.^46^ has been carried out for the case study area, and the comparison between the results obtained using the different formulations has been described.

## Method

This section provides details on the new proposed CVI formulation describing the variables used, the ranking score and the mathematical formulation applied to construct the synthetic index. This section also describes the statistical and sensitivity analysis carried out for the preparation of this work, and the application and comparison of other CVI formulations present in the literature for the case study area.

### CVI formulation

A stretch of coast is divided into a number of transects (or cross-sectional profiles of the beach) to assess its vulnerability. Each transect is characterized by a control area 0.5 km wide. A relative vulnerability score is assigned to each variable based on the potential magnitude of its contribution to physical changes on the coast^2–4^. Variables are ranked on a linear scale from 1 to 5 in order of increasing vulnerability. A mathematical formulation is used to construct the synthetic index and the CVI values are classified into four different groups using percentiles of the distribution as limits.

The newly proposed CVI formulation uses the variables described in the following table (Table 2).

**Table 2:** Variables used in the proposed CVI formulation.

Type of variables	#	Variables	Remarks
Geologic	1	Geomorphology	Proposed by^4^ and already considered in the previous authors’ CVI^27^
	2	Coastal slope (%)	Proposed by^4^ and already considered in the previous authors’ CVI^27^
	3	Shoreline erosion/accretion rates (m/year)	Proposed by^4^ and already considered in the previous authors’ CVI^27^
	4	Emerged beach width (m)	Already considered in the previous authors’ CVI^27^
	5	Dune	Redefined considering the dune width, already used in the previous authors’ CVI^27^, and the type of dune
Hydro-physical process	6	River discharge (m^3^/s)	New variable added in the present work
	7	Relative sea-level change (mm/year)	Proposed by^4^ and already considered in the previous authors’ CVI^27^
	8	Mean significant wave height (m)	Proposed by^4^ and already considered in the previous authors’ CVI^27^
	9	Mean tide range (m)	Proposed by^4^ and already considered in the previous authors’ CVI^27^
Vegetation	10	Vegetation behind the back-beach	Redefined considering the width of vegetation behind the beach, already used in the previous authors’ CVI^27^, and the type of vegetation
	11	Coverage of *Posidonia oceanica* (%)	Redefined considering not only its presence/absence but also the bottom coverage of meadows

For the CVI method previously proposed by the authors^27^, a table reporting the variables used and the ranking scores is included as Supplementary materials (Table S.1).

The integration of the variables “Dune” and “Vegetation behind the back-beach” is aimed at allowing a more in-depth evaluation of the ability of the coastal system to dissipate wave energy and better respond to extreme events. The “Dune” variable considers both aspects of dune width and dune type. Dune width, already considered in the previous formulation^27^, is important for the conservation of the coastal zone, as it increase its resilience^47–50^; the temporal dominance of the wave collision regime, wherein volume loss from the dune occurs through dune retreat without overtopping, suggests that dune width must be considered an important factor of coastal defence^51^. Although it constitutes one of the most important factors of resistance to wave attacks and erosion, dune systems offer a different degrees of resistance/resilience in relation to their consolidation and typology^52,53^. Therefore, this variable combines these two aspects to characterize the overall role of dunes in coastal protection.

Similarly, the variable “Vegetation behind the back-beach” considers the two aspects of width of the vegetation behind the back-beach and type of vegetation. The width of the vegetation, already considered in the previous formulation^27^, is related to the coastal defence against storm events and inundation; the type of vegetation is also important, as it plays a crucial role in the defence of coastal areas and infrastructures along the coast by decreasing wave run-up and decreasing sand loss by wave backwash^54^. Furthermore, the size, shape and stability of dunes are strongly controlled by vegetation cover. Therefore this variable combines these two aspects to take into account the overall effects of vegetation on wave energy dissipation and erosion reduction.

In the same way, the variable “Coverage of *Posidonia oceanica*” allows us to take into account the implications of these meadows for coastal defence. The *Posidonia oceanica* meadows constitute an effective barrier for coastal defence from erosion due to both seabed stabilization and wave motion damping^55,56^. Consequently, the presence of these meadows and their degree of coverage are both relevant, as they affect on the protection capacity of the beaches.

Finally, new variable, “River discharge”, it has been added considering that water and sediments discharged from rivers into the sea impact sedimentation processes and dynamic balance, particularly in low-lying and sedimentary coasts^57^. However, it should also be highlighted that anthropogenic factors, such as, river basin regulation works (especially dams), determine changes in sediment transport processes and water discharge. In such cases, the potential role of this variable may be limited and therefore require a more careful evaluation of local features and peculiarities in the specific study area.

Below is a description of the variables and relative ranking scores.

The geological variables are:

- *Geomorphology* This variable expresses the relative erodibility of different landform types (e.g., rocky cliffs, and sandy beaches) along the coast and requires information on the spatial distribution of landform types and their stability^4,27^. Ranges of vulnerability scores are defined according to the relative susceptibility of a given landform to physical change and are those proposed by^4^; the ranking score assigns the lower vulnerability score to rocky and cliff coasts and the higher vulnerability score to landform types such as barrier beaches, sand beaches, and deltas.
- *Coastal slope* This variable is an indicator of the relative vulnerability to inundation and of the potential rapidity of shoreline retreat^27^. Its ranking scores have been defined considering previous studies carried out for the Mediterranean coast^18,41,42^. In particular, the ranges used here refer to those proposed by López Royo et al.^18^. Considering inundations, storm surges and associated land losses, a coast characterized by a gentle slope is considered more vulnerable than a coast with a steeper slope. Consequently, for the case study area, the highest score (very high vulnerability) is assigned at coastal slope < 2% and the lower score value (very low vulnerability) for coastal slope > 12%. Low vulnerability is considered on slopes 4–2%, moderate vulnerability for slopes 8–4%, and high vulnerability for slopes 12–8%.
- *Shoreline erosion/accretion rates* This variable assesses the state of erosion or accretion^27^. Ranking scores have been defined considering those proposed by Karymbalis et al.^42^. In particular, shoreline change characterized by erosion rates <  − 1.5 m/year is considered very high vulnerability, while accretion rates > 1.5 m/year are considered very low vulnerability. Low vulnerability is considered for accretion rates of 1.5–0.5 m/year, moderate vulnerability for values between 0.5 and − 0.5 m/year, and high vulnerability for values between − 0.5 and − 1.5 m/year.
- *Emerged beach width* Ranges of vulnerability scores are defined in consideration of characteristics endemic to the Italian and Mediterranean areas^58^ and of the fact that a wider beach has a greater ability to dissipate wave energy and to better defend against extreme events. Therefore, for the case study area, the vulnerability score of emerged beach width > 100 m is considered very low. Beach widths from 50–100 m are considered low vulnerability, 25–50 m moderate vulnerability, 25–10 m high vulnerability, and < 10 m very high vulnerability.
- *Dune* A relative score is assigned separately to both the dune width and the type of dune.

For the dune width, the relative scores are defined considering that wide dunes constitute a greater defence factor than narrow dunes during storms and extreme events. Therefore, the relative score varies from 1 (dune width > 100 m) to 5 (dune width <25 m) according to class intervals of 25 m.

Regarding the type of dune, the classification described in NSW (Department of Land and Water Conservation)^52^ is used as a reference and considers three typical features of a dynamic beach system: incipient dune, foredune and hind dune. In particular, in the absence of a dune system the highest relative score is assigned (score 5), while score values equal to 4, 3 and 2 are assigned in the case of incipient dunes, foredunes and hind dunes, respectively.

A mathematical formulation is used to combine the relative scores assigned separately to dune width and type of dune, as described below:

1 $$D =\bigg \lfloor \frac{{D_{DW } + 5/4(D_{TD} - 1)}}{2} \bigg \rceil $$

where *D*_*DW*_ and *D*_*TD*_ are the relative scores assigned to dune width and type of dune, respectively, and *D* is the total value associated with the variable. According to this mathematical formulation the value of *D* varies from 1 to 5, and corresponds to the same ranking score vulnerability for the “Dune” variable.

The hydro-physical process variables are:

- *River discharge* This variable is added to consider the aspect of discharge and sediment transport that affects the morphodynamics of coastal systems^57,59^. The ranking scores, are defined considering the peculiar characteristics of the Calabrian territory; they are applied to the following categories: stream (river discharge less than 1 m^3^/s), small river (river discharge between 1 m^3^/s and 5 m^3^/s), medium river (river discharge between 5 m^3^/s and 20 m^3^/s) and large river (river discharge greater than 20 m^3^/s). Considering that CVI provides a numerical basis for comparing transects to identify those where vulnerability may be relatively greater, the presence of a river in the transect is a factor that can contribute into reducing vulnerability in the area. Therefore, the highest vulnerability score is assigned in the absence of rivers/streams, while high, medium, low and very low vulnerability scores are assigned in the case of streams, small rivers, medium rivers and large rivers, respectively. River discharge is used because it is closely associated with sediment transport^59^.
- *Relative sea level change* This variable is derived from the time series of sea level records at each tide gauge station along the coast; it includes both eustatic sea-level rise and regional sea-level rise due to isostatic and tectonic adjustments of the land surface. Relative sea-level change data are a historical record, and thus show change for only a recent time scale^4^. In this work ranking scores are defined in agreement with those proposed by Karymbalis et al.^42^. The rate of relative sea-level rise is ranked considering the values < 1.8 mm/yr as very low vulnerability, the values 1.8–2.5 mm/year as low vulnerability, the values 2.5–3.0 mm/year as moderate vulnerability, the values 3.0–3.4 mm/year as high vulnerability and the values > 3.4 mm/year as very high vulnerability.
- *Mean significant wave height* This variable is used as a proxy for wave energy, which drives the coastal sediment budget^4^. The wave energy increases as the square of the wave height, and therefore, the vulnerability score assumes an increasing value as the height of the waves increases. For the case study area, the ranking scores are those proposed by Karymbalis et al.^42^ and vary from very low (< 0.3 m) to very high (> 1.2 m) according to class intervals of 0.3 m.
- *Mean tide range* This variable is linked to both permanent and episodic inundation hazards. Some researchers^3,43^ consider that coastline with a large tidal range must be assigned a high vulnerability classification, while microtidal coasts receive a low vulnerability rating. The main reasoning is that a large tidal range delineates a broad zone of intertidal area that will be most susceptible to inundation following long-term sea-level rise. Moreover, a large tidal range is associated with strong tidal currents that influence coastal behaviour. Instead, other studies adopt the opposite assumption. In this work, the vulnerability ranges are defined in accordance with those proposed by Thieler and Hammar-Klose^4^. This assumption is based on the concept that, in general, microtidal (tide range < 2.0 m) and macrotidal (tide range > 4.0 m) are characterized by high and low risk, respectively. The reasoning is based primarily on the potential influence of storms on coastal evolution, and their impact relative to the tide range. For example, on a tidal coastline, there is only a 50 percent chance of a storm occurring at high tide. Thus, for a region with a 4.0 m tide range, a storm with a 3 m surge height is still up to 1 m below the elevation of high tide for half a tidal cycle. A microtidal coastline, on the other hand, is essentially always “near” high tide and therefore always at the greatest risk of inundation from storms^4^. It is worth highlighting that this assumption has also been used for other studies present in the literature^5,6,10,17,41^. The Mediterranean area is a microtidal environment and the coast of Calabria has a tidale range < 1 m. Rankings scores vary from very low (> 6 m) to very high (< 1 m)

The vegetation variables are:

- *Vegetation behind the back-beach* Regarding the width of vegetation behind the back-beach, it should be noted that vegetation is determined by clear and obvious signs of flora, indicated by the green area behind an emerged beach (back-beach); it may be interrupted in places where it intersects with infrastructures such as roads, and houses.^27^; Its relative score is defined to indicate that the degree of coastal vulnerability decreases with the increase in width of vegetation behind the back-beach due to the vegetation influence on sediment transport and wave impact. It should be noted that in the case of sparse vegetation a careful evaluation of the specific characteristics of the area must be considered; therefore, in this case it is necessary to carefully analyse the special coverage and prevailing vegetation and evaluate whether to assign a more precautionary higher vulnerability score. The relative scores assigned considering the width of the vegetation are the following: width > 400 m, relative score 1; width from 400 to 200 m, relative score 2; width from 200 to 100 m, relative score 3; width from 100 to 50 m, relative score 4; width < 50 m, relative score 5. Regarding the type of vegetation, three types of vegetation are considered in relation to their different actions of defence and protection of the coast from inundation: primary species, secondary species and tertiary species. Primary species consist of herbs, grasses and creepers; secondary species consist of shrubs, ground plants and short-lived trees; and tertiary species consist of long-lived trees^52^. The relative scores are defined considering the three types of vegetation. The highest relative score is assigned in the absence of vegetation and decreasing values for primary species (relative score 4), secondary species (relative score 3) and tertiary species (relative score 2) in relation to their different action of coastal defence.
  A mathematical formulation is used to combine the relative scores assigned separately to the width of vegetation behind the back-beach and the type of vegetation, as described below:
  2 $$V = \bigg \lfloor \frac{{V_{DV } + 5/4(V_{TV} - 1)}}{2}\bigg \rceil$$
  where *V*_*DV*_ and *V*_*TV*_ are the relative scores assigned to the width of vegetation behind the back-beach and the type of vegetation, respectively, and *V* is the total value associated with the variable. According to this mathematical formulation, the value of *V* varies from 1 to 5, which correspond to the same ranking score vulnerability for the variable.
- *Coverage of Posidonia oceanica* This variable takes into account the presence of these meadows and their degree of coverage. *Posidonia oceanica* is a marine phanerogam endemic to the Mediterranean basin that forms extended meadows along its coasts in a bathymetric surface to 0–40 m depth in clear waters^60^. It plays a crucial role in the physical equilibrium of a large portion of the Mediterranean coasts. Although its benefits occur mainly below seagrass meadows, where it stabilizes the sea bottom by reducing sediment mobilization, the presence of *Posidonia oceanica* has effects on coastal protection, stabilization of the shoreline and beach morphology^55,56,60–67^; furthermore, studies have documented the effect of reducing wave energy. Large-scale flume experiments on artificial *Posidonia oceanica* meadows and studies using field data have been carried out to measure wave attenuation and energy dissipation in intermediate and shallow waters while also considering seagrass density. These studies confirm the attenuation, transmission and energy dissipation of waves induced by *Posidonia oceanica* meadows^68–71^.

Therefore, this variable takes into account these positive effects on coastal protection related to the presence of coverage of *Posidonia oceanica*. In the previous CVI formulation proposed by the authors^27^, *Posidonia oceanica* was assessed only in relation to its presence/absence; in this work *Posidonia oceanica* is evaluated considering its bottom coverage, which affects its ability to mitigate damage caused by storm surges and inundations. Bottom coverage is expressed as a percentage of the seabed surface covered by living plants. The ranges have been defined by the percentage of coverage of these meadows with respect to the seabed area considering cells of 0.5 km x 1 km. In the absence of *Posidonia oceanica* meadows the highest vulnerability score is assigned, while high, medium, low and very low vulnerability values are assigned using coverage percentages <25%, 50–25%, 75–50%, and >75%, respectively.

Table 3 shows the range of vulnerability for the 11 variables.

**Table 3:** Ranges of vulnerability scores for the variables.

Type of variables	Variables	Score					Reference/method
		1 Very low	2 Low	3 Moderate	4 High	5 Very-high	
Geologic	Geomorphology	Rocky, cliffed coasts	Medium cliffs, indented coasts	Low cliffs, alluvial plains	Cobble beaches, estuary, lagoon	Barrier beaches, sand beaches, salt marsh, mud flats, deltas, coral reefs	Thieler and Hammar-Klose^4^
	Coastal slope (%)	> 12	8–12	4–8	2–4	< 2	López Royo et al.^18^
	Shoreline erosion/accretion rates (m/year)	> (+ 1.5)	(+ 1.5)–(+ 0.5)	(− 0.5)–(+ 0.5)	(− 0.5)–(− 1.5)	< (− 1.5)	Karymbalis et al.^44^
	Emerged beach width (m)	> 100	50–100	25–50	10–25	< 10	Pantusa et al.^27^
	Dune	1	2	3	4	5	Mathematical formulation combining the relative scores of dune width and type of dune
Hydro-physical process	River discharge (m3/s)	Large river	Medium river	Small river	Stream	Absent	River discharge
	Relative sea-level change (mm/year)	< 1.8	1.8–2.5	2.5–3.0	3.0–3.4	> 3.4	Karymbalis et al.^44^
	Mean significant wave height (m)	< 0.3	0.3–0.6	0.6–0.9	0.9–1.2	> 1.2	Karymbalis et al.^44^
	Mean tide range (m)	> 6.0	4.0–6.0	2.0–4.0	1.0–2.0	< 1.0	Thieler & Hammar-Klose^4^
Vegetation	Vegetation behind the back-beach	1	2	3	4	5	Mathematical formulation combining the relative scores of width of vegetation behind the back-beach and type of vegetation
	Coverage of *Posidonia oceanica* (%)	> 75	50 -75	25 – 50	< 25	Absent	Percentage

The CVI is obtained by the square root of the product of the vulnerability scores assigned to each variable divided by the total number of variables:

3 $$CVI = \sqrt {\left( {a \cdot b \cdot c \cdot d \cdot e \cdot \dot{f} \cdot g \cdot h \cdot i \cdot j \cdot k} \right)/11}$$

where a = geomorphology, b = coastal slope, c = shoreline erosion/accretion rates, d = emerged beach width, e = dune, f = river discharge, g = relative sea-level change, h = mean significant wave height, i = mean tide range, j = vegetation behind the back-beach, k = coverage *of Posidonia oceanica.*

The CVI values obtained considering the vulnerability associated with each variable according to the formulation described in Eq. (3) have been divided into four categories of vulnerability:

- Low vulnerability (green).
- Moderate vulnerability (yellow).
- High vulnerability (orange).
- Very-high vulnerability (red).

These categories are obtained by considering the 25th, 50th and 75th percentiles of the distribution of the CVI values across the transects^4^.

### Statistical and sensitivity analysis

The variance inflation factor (VIF) is used to measure the severity of multicollinearity in regression analysis^72^. A VIF value > 10 is considered an indicator that the collinearity among the variables is too high and t the regression output is unreliable; therefore, several studies recommended a value of 10 as the maximum acceptable VIF limit^73–75^.

Considering overall vulnerability, Pearson’s product-moment correlation coefficient values are computed to show the most influencial group of variables.

To evaluate the response of the index to changes in the score ranking, aggregation formulation and vulnerability classification method, a local sensitivity analysis is performed.

In particular, the categorical scale that uses equal classes defined between min–max values of the distribution and the percentile method is considered to compute different score rankings for some variables. Correlation coefficients are computed to compare the output of scores for each considered variable. A high correlation denotes that the variable is lightly sensitive to changes in the ranking scoring, while a low correlation denotes that the variable is highly sensitive to change. First, sensitivity analysis is carried out considering each variable separately, and subsequently, sensitivity analysis is also carried out considering the variations in overall CVI results using the two score ranking simultaneously for all considered variables. Regarding the ranking scores analysed, it is worth noting that the use of equal intervals and percentiles of the distribution values, representing the relative vulnerability of the coast, are considered as they are the most commonly used formulations in categorical scale methods and benchmarking approaches.

Regarding the aggregation formulation, sensitivity analysis is performed by testing two different formulations: the average sum of squares and the modified product mean.

Finally, sensitivity analysis is performed on the overall CVI vulnerability classification method using equal classes between min–max values of the distribution as it is a method also used in several applications.

Statistical and sensitivity analyses are performed using the Octave 5.2.0 software (https://ocatve.org).

### Application and comparison of three methods for the case study area

It should be noted that there is no a unique approach to assessing coastal vulnerability, and it might be useful to select and customize the variables considering the site-specific conditions to make the overall CVI results truly useful for vulnerability analysis and intervention/investment planning. Similarly, according to Koroglu et al.^19^, it might be useful to consider site or region-specific ranking categories to develop reliable tools for local coastal management.

Therefore, in this work, a comparison of the results of the application for the case study area considering the previous CVI formulation proposed by the authors^27^, the CVI method proposed by Ružić et al.^45^, and the CVI formulation proposed by Palmer et al.^46^, is carried out.

Regarding the CVI method proposed by Ružić et al.^45^, the variables considered are: geologic fabric, coastal slope, beach width, significant wave height, land use. The CVI is computed as the square root of the vulnerability scores assigned to each variable product divided by the total number of variables, emphasizing the geological fabric’s importance:

4 $$CVI_{{{\text{Ruzic et al}}.{ }}} { } = \sqrt {\left( {a^{2} \cdot b \cdot c \cdot d \cdot e} \right)/6}$$

where a = geologic fabric, b = coastal slope, c = beach width, d = significant wave height, e = land use. The index gives priority to the geological fabric considered the most important factor of coastal negative changes, and it was developed to analyze an area characterized by complex geomorphology^46^.

Regarding the method proposed by Palmer et al.^46^, the physical variables used are: beach width, dune width, distance to 20 m isobath, distance of vegetation behind the back beach, percentage of outcrop. The relative physical coastal vulnerability is computed as follows:

5 $$RelativeCVI = a + b + c + e + f + g$$

where a = beach width, b = dune width, c = distance to 20 m isobath, d = distance of vegetation behind the beach, e = percentage of outcrop, f = additional weighting of highly vulnerable sites, g = additional weighting if cell intersects an estuarine area. The index refers to the relative coastal vulnerability of coast to erosion and extreme weather events, and it was validated by the comparison to historical data from past coastal erosion event impact sites^47^.

For these two CVI methods, a table reporting the variables used and the ranking values is included as Supplementary materials (Table S.2).

## Case study

### Description of the case study area

**Figure 1:**
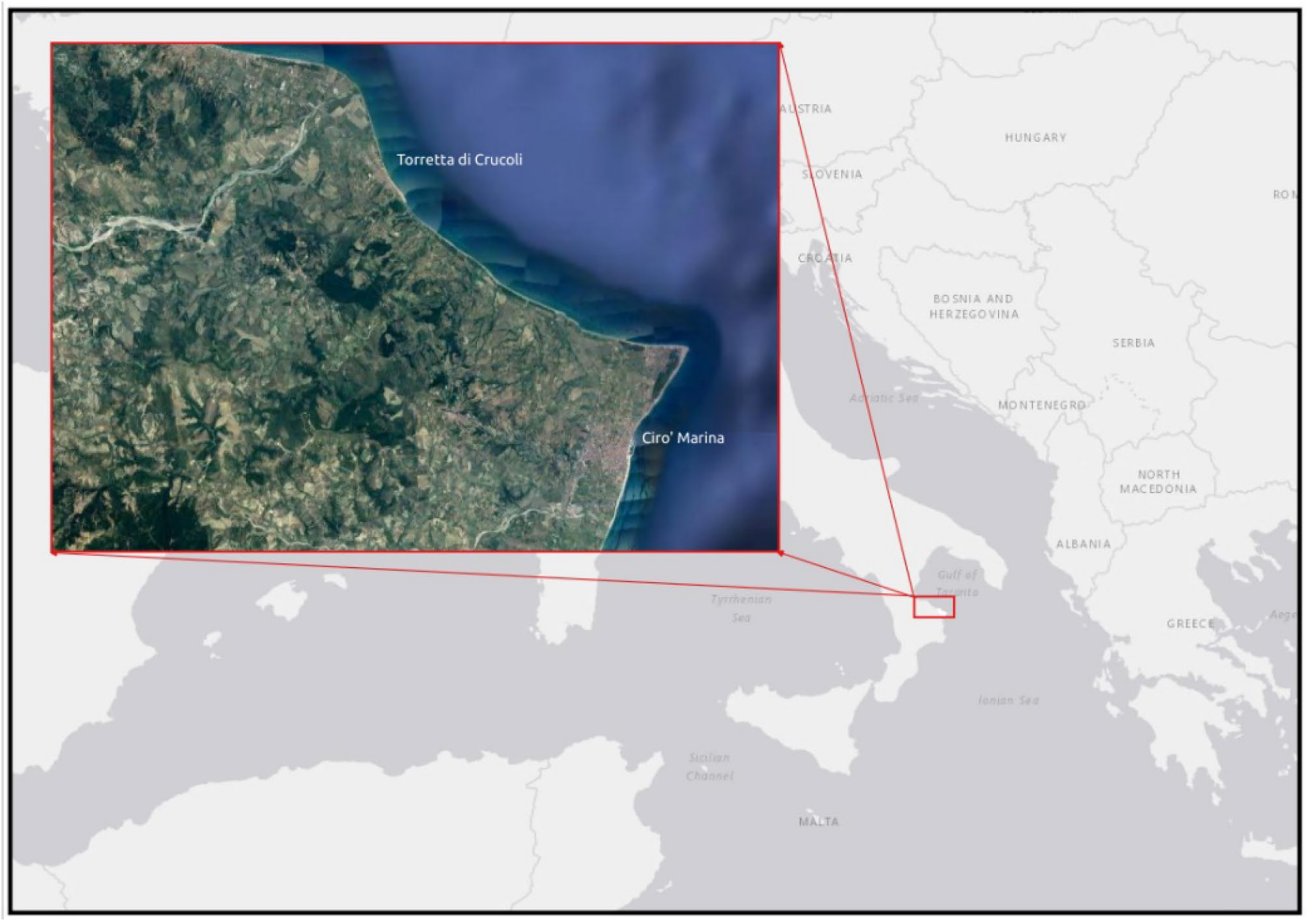
Case study area. Torretta di Crucoli – Cirò Marina, Calabria region, Italy. The map was created using QGIS 3.29 software (https://qgis.org).

The study area is located in the province of Crotone, Calabria region, southern Italy, and extends for a length of 20 km (Fig. 1).

This area is representative of a typical Mediterranean coastline and has two large urban settlements that have been significantly developed since the start of the second half of the twentieth century due to the growth of the agricultural and fishing sectors, and the related processing industries. Craft and recreational activities have also arisen, linked to seaside tourism. In this coastal stretch, there is an important railway affected by threats of service disruption during storm events. The typology of coastal beaches is low-lying sandy beach characterized by fine and medium-sized sand granulometry.

**Figure 2:**
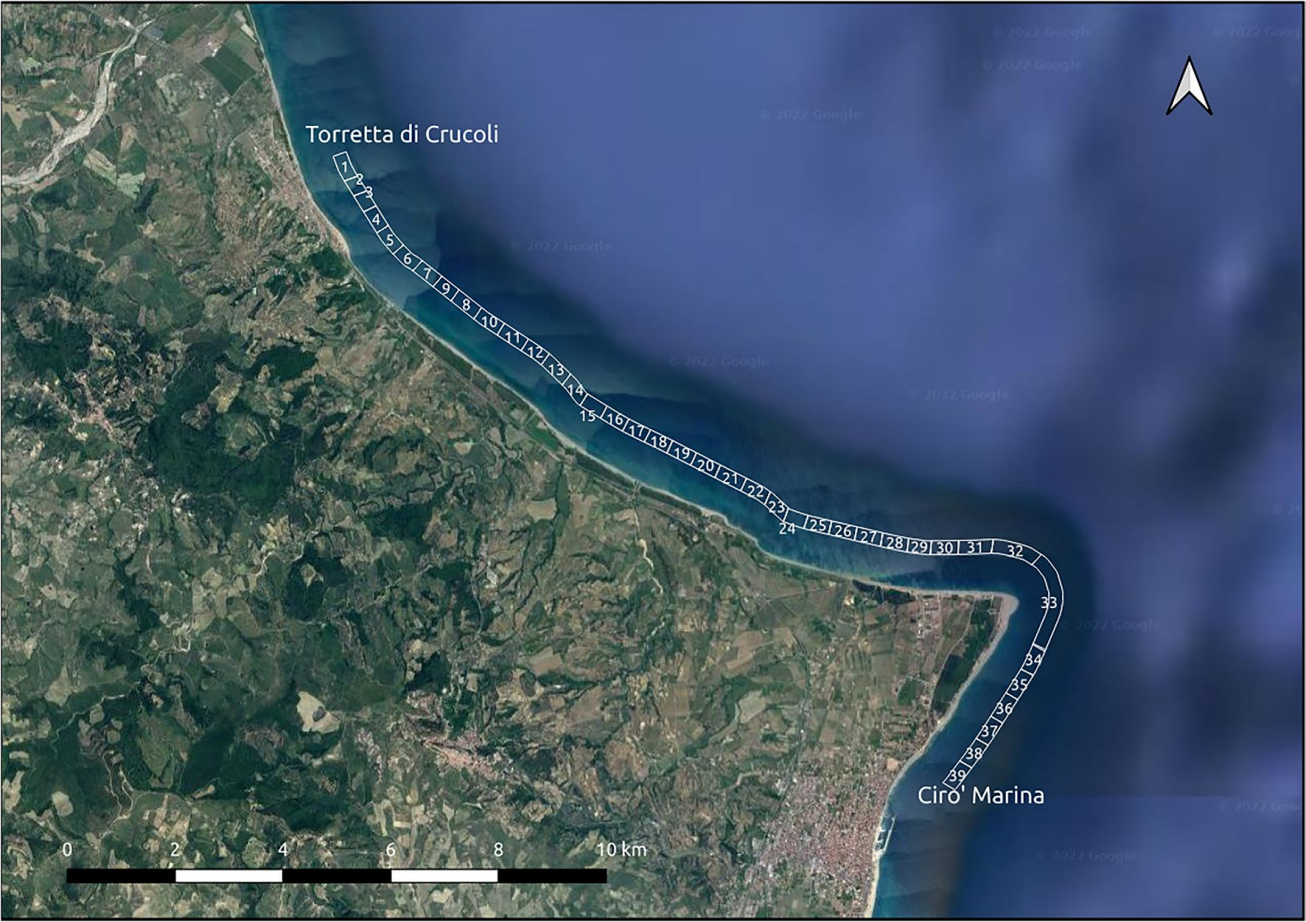
Case study area and related transects. The map was created using QGIS 3.29 software (https://qgis.org).

The area has been divided into 39 transects with a width of approximately 500 m, mainly consisting of a sandy beach with dune systems, among which there are areas of important naturalistic interest, such as the “Dunes of Marinella”, which has constituted a Site of Community Importance (SCI) since 1999, as defined in the European Commission Habitats Directive (92/43/EEC). This area represents one of the largest (up to 800 m) coastal dune/paleodune systems in Calabria^76^. This system is characterized by well-sorted, medium to fine brown sands, sometimes reddish in color. Hydrography of the area consists mainly of ditches and streams. The coastal slope is low, and most of the transects are in a state of erosion. The relative sea-level change is 4 mm/year, the mean significant wave height is 1.12 m, and the tide regime is microtidal. The first 4 transects consist of sandy beaches and are located in the town of Torretta di Crucoli; the following 3 transects have incipient dunes and foredunes and areas where the dune system has been damaged by buildings. The next 13 transects are in the “Dunes of Marinella” area and consist mainly of hind dunes, reaching to the “Madonna di mare” locality. The following 6 transects are characterized by incipient dunes. The next 11 transects are characterized by wider beaches and hind dunes, though the dunes of three transects have been damaged over the years. The last 2 transects are sandy beaches near the town of Cirò Marina. Figure 2 shows the case study area and related transects.

### Data

The data sources used to define all the variables used for the CVI formulation are described in the following table (Table 4).

**Table 4:** Data sources.

Variable	Data summary	Data source
Geomorphology	Map data (DTM) and lithological map (0.5 km grid cell)	Geoportal of Calabria Region (http://geoportale.regione.calabria.it/opendata)
Coastal slope	Regional bathymetry and map data (DTM) (Grid extending 5 km landward and seaward of the shoreline—elevation at 1 m vertical resolution for 8 m grid cells)	Geoportal of Calabria Region (http://geoportale.regione.calabria.it/opendata)
Shoreline erosion/accretion rates	Regional orthophotos (1954 -2018)	Geoportal of Calabria Region (http://geoportale.regione.calabria.it/opendata)
Emerged beach width	Regional orthophotos	Geoportal of Calabria Region (http://geoportale.regione.calabria.it/opendata)
Dune	Dune width: regional orthophotos; Type of dune: visual inspection and previously studies carried out in the area	Geoportal of Calabria Region (http://geoportale.regione.calabria.it/opendata) Uzunov et al. (2013)^76^; Uzunov et al. (2009)^77^
River discharge	River discharge data	Authority Basin of Calabria region (http://old.regione.calabria.it/abr/index.php?option=com_content&task=view&id=364&Itemid=113)
Relative sea-level change	Trends in Sea level rise	Mediterranean by National Oceanic and Atmospheric Administration, NOAA (https://www.star.nesdis.noaa.gov/socd/lsa/SeaLevelRise/)
Mean significant wave height	Wave information (Crotone wave buoy)	National wave metric network (ISPRA—Institute for Environmental Protection and Research)
Mean tide range	Tide range information	European Environmental Agency EEA data-base (https://www.star.nesdis.noaa.gov/socd/lsa/SeaLevelRise/);
Vegetation behind the back-beach	Regional orthophoto	Geoportal of Calabria Region (http://geoportale.regione.calabria.it/opendata)
Coverage of *Posidonia oceanica*	Regional orthophoto and previously study	Geoportal of Calabria region (http://geoportale.regione.calabria.it/opendata); EEA(https://nature-art17.eionet.europa.eu/article17/reports2012/habitat/summary/?period=5&group=Coastal+habitats&subject=1120&region=MMED)

### Results statistical and sensitivity analysis results

“Geomorphology”, “Relative sea-level change”, “Mean significant wave height”, and “Mean tide range” assume constant values for the case study area. Preliminarily, these variables were excluded; then, the VIF values are computed, and were within 10; The obtained VIF values are the following: coastal slope (1,25); shoreline erosion/accretion rates (1,15); emerged beach width (1,18); dune (3,02); river discharge (1,09); vegetation behind the back-beach (2,92); coverage of *Posidonia oceanica* (1,57).

**Figure 3:**
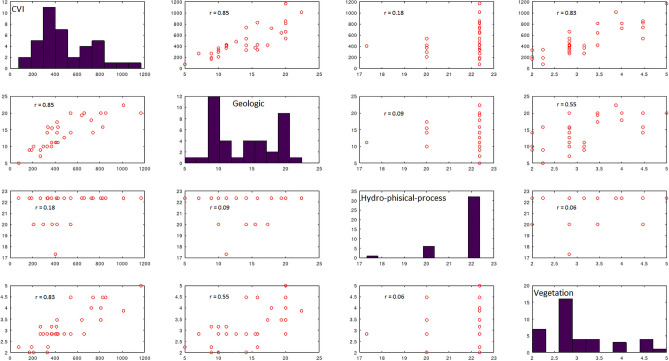
The scatter plot shows the results of Pearson’s product-moment correlation considering the three groups of variables and the overall CVI value with the correlation coefficient value (r) highlighted.

Regarding statistical analysis, in addition to the VIF calculation described above, Pearson’s product-moment correlation coefficient values were computed to identify the most influential group of variables on the overall CVI value (Fig. 3). The correlation coefficient values between the overall CVI values and the variables of the three groups (Geological, Hydro-physical process and Vegetation) show that the Geological and Vegetation variables, which are characterized by the greatest spatial variability, are characterized by a high relationship (r = 0.851 and r = 0.826, respectively); therefore, these two groups have the most influence on the overall CVI values, while the Hydro-physical process variables have less influence on the physical vulnerability of the coast (r = 0.180).

Regarding sensitivity analysis on ranking score, variables characterized by a constant value for all transects and those that included qualitative data were excluded. As previously mentioned, two different ranking scores have been tested: categorical scores using equal classes defined considering the min–max values of the distribution, and the percentile rank (20th, 40th, 60th, 80th).

The following table (Table 5) shows the correlation coefficients calculated to compare variations in the score ranking outputs for each considered variable. Furthermore, it shows the correlation coefficient of the overall CVI output, simultaneously considering all the variables tested and their ranking scores. The table also shows the correlation coefficients computed to compare variations in the aggregation method and in the vulnerability categories rank.

**Table 5:** Correlation coefficient values—sensitivity analysis on ranking score, aggregation formulation and vulnerability classification method.

	**Correlation coefficient (r)**	
	**Equal classes (Min–Max)**	**Percentiles rank**
**Ranking score**		
Coastal slope	0.831	0.860
Shoreline erosion/accretion rates	0.734	0.815
Emerged beach width	0.761	0.781
Dune	0.895	0.937
Vegetation behind the back- beach	0.909	0.933
Coverage of *Posedonia oceanica*	0.538	0.606
CVI	0.366	0.405

	**Correlation coefficient (r)**	
**Aggregation method**		
Average sum of squares	0.863	
Modified product mean	0.950	
**Vulnerability classification method**		
Equal classes (Min–Max)	0.898	

### Results of the CVI application

As mentioned above, for the “Geomorphology” variable, the value is constant for all transects at very high vulnerability, while for “Coastal slope” the values vary between 0.94% (min) and 4% (max) with transects at high and very high vulnerability scores. Regarding “Shoreline erosion/accretion rates”, most transects are at very high and high vulnerability. Only two transects are at very low vulnerability. Almost all the transects are in a state of erosion, with a minimum retreat value of approximately 0.4 m and a maximum value of approximately 7 m. Regarding the other transects, the minimum coastal accretion value is approximately 0.5 m while the maximum value is approximately 15 m.

“Emerged beach width” includes very low vulnerability and high vulnerability, and it varies between the minimum value of approximately 22 m and the maximum value of approximately 204 m with a prevalence of transects at moderate vulnerability value, characterized by a beach width of about 50 m.

Regarding the variable “Dune”, it should be highlighted that dune width varies from a minimum of 91.5 m to a maximum of 470 m, while the dune system is present in 32 transects, 17 of which are of hind dune type, 8 of which are foredune types and the remaining are incipient dunes. The overall “Dune” is classified as very-low to very high vulnerability.

Regarding the variable “River discharge”, most transects are at very high vulnerability, few have values of high vulnerability and one transect is within the moderate vulnerability score range; the surface water circulation of the study case area mainly consists of drainage ditches, except for 7 transects characterized by torrential rivers, 6 of which are classified as “stream” and one as “small river”.

“Relative sea-level change” is constant for all transects (4 mm/year) at very high vulnerability, “Mean significant wave height” is constant at high vulnerability (1.12 m), while “Main tide range” is constant at very high vulnerability (microtidal).

Regarding the variable “Vegetation behind the back-beach”, the maximum width of vegetation value is approximately 1100 m with an average value of approximately 350 m, and most of the transects are characterized by tertiary vegetation, except in the transects near the towns and for the transect without the dune system. Overall, this variable is classified as very low to very high vulnerability.

Finally, the “Coverage of *Posidonia oceanica*” includes transects at high vulnerability, few transects at very high vulnerability and one transect that is within the moderate vulnerability score category; in particular, *Posidonia oceanica* is absent in 9 transects, and in the other transects the maximum coverage is 27% with average coverage values of approximately 5%. The vulnerability values are mainly high, followed by very-high vulnerability values.

**Figure 4:**
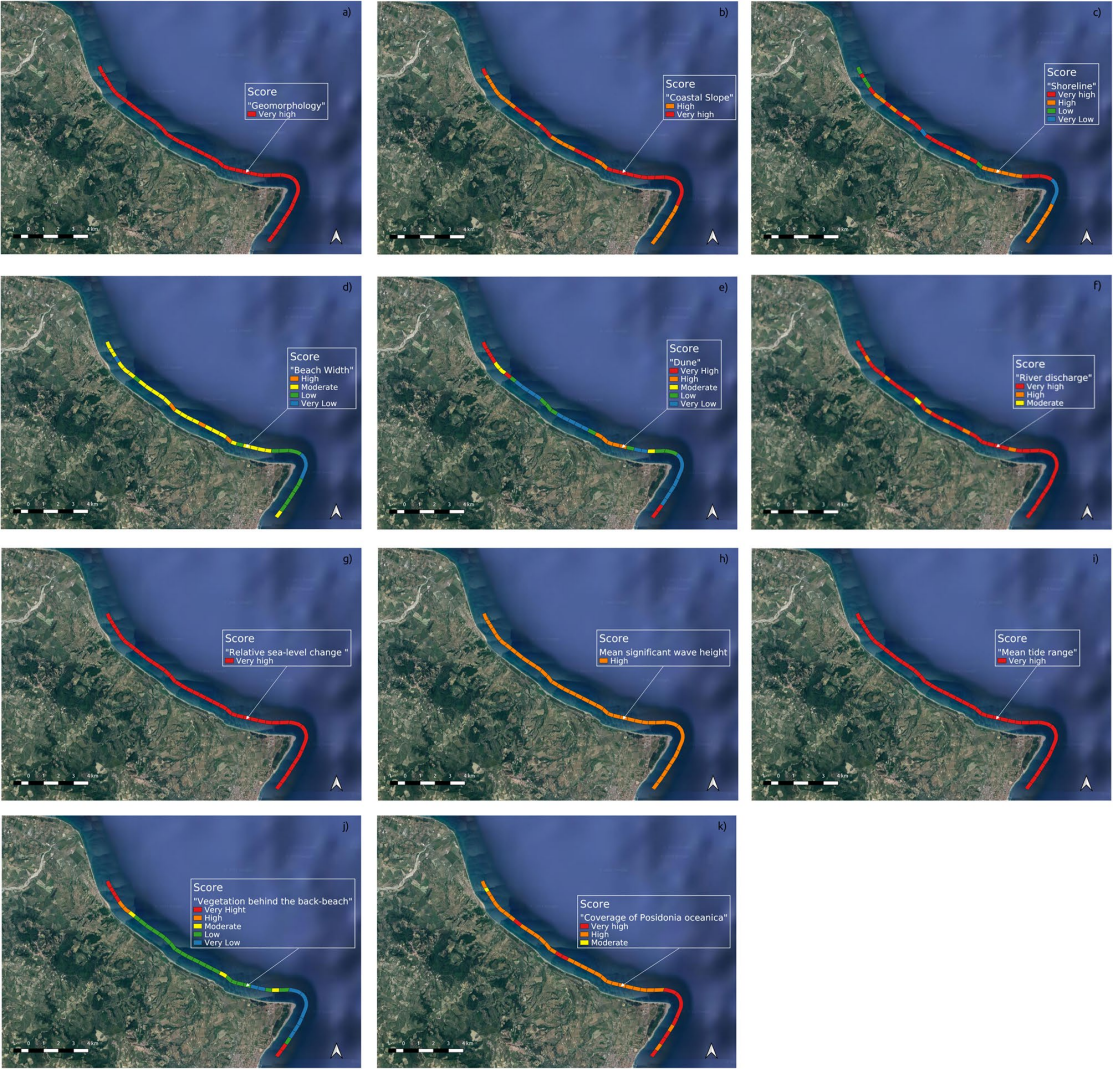
Score value maps for each variable: (**a**) geomorphology, (**b**) coastal slope, (**c**) shoreline erosion/accretion rates, (**d**) emerged beach width, (**e**) dune, (**f**) river discharge, (**g**) relative sea-level change, (**h**) mean significant wave high, (**i**) mean tide range, (**j**) vegetation behind the back-beach, (**k**) coverage of *Posidonia oceanica*. The map was created using QGIS 3.29 software (https://qgis.org).

The following figure (Fig. 4) shows the score value maps of the transects based on each variable.

The estimated CVI values vary between 75.38 (min) and 1167.75 (max). The CVI mean is 485.01 and the median is 412.86. Table 6 shows the vulnerability category.

**Table 6:**
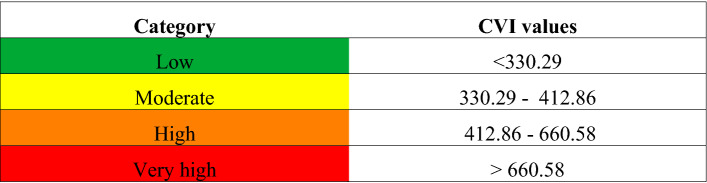
Vulnerability category.

**Figure 5:**
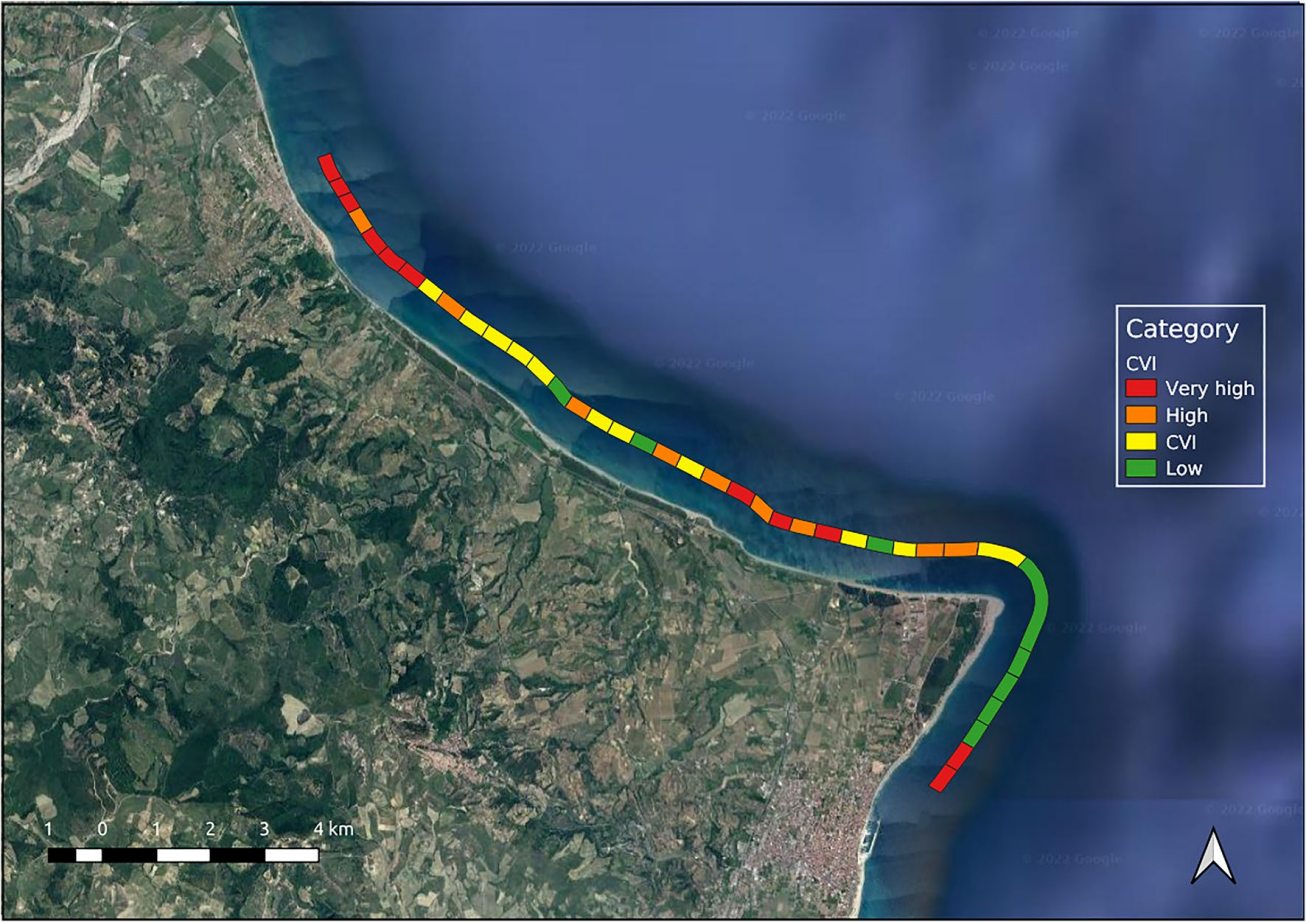
CVI category for each transect. The map was created using QGIS 3.29 software (https://qgis.org).

Figure 5 shows the obtained CVI category for each transect.

A table describing the vulnerability score values associated with each variable and the estimated CVI value for each transect is included in the Supplementary materials (Table S.3).

### Results of the comparison of three methods for the case study area

Regarding the comparison with the previous formulation proposed by the authors^27^, the results obtained have been compared to highlight the effects of the added variables to better characterize the case study area.

The aim of a comparison with the two other methods is to test the sensitivity of these indices regarding the characteristics of the study case area and to assess whether the newly proposed formulation is better suited to the specific characteristics of the case study coastline, a typical low-lying coast of the Mediterranean. The CVI method proposed by Ružić et al.^45^ was developed to analyse a stretch of the Mediterranean coast, the Croatian eastern Adriatic coast; this coast is characterized by coastal retreat, slope instability and erosion conditions, similar to the case study area described in this paper. The CVI method proposed by Palmer et al.^46^, was chosen because it uses some variables similar to those proposed in this paper and has been applied for a coastline mainly consisting of low-lying sandy beaches.

**Figure 6:**
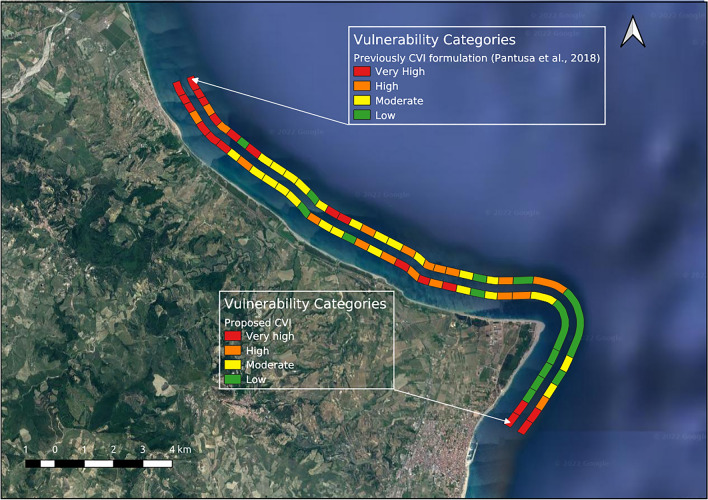
Proposed CVI formulation and previously CVI formulation^27^. The map was created using QGIS 3.29 software (https://qgis.org).

**Figure 7:**
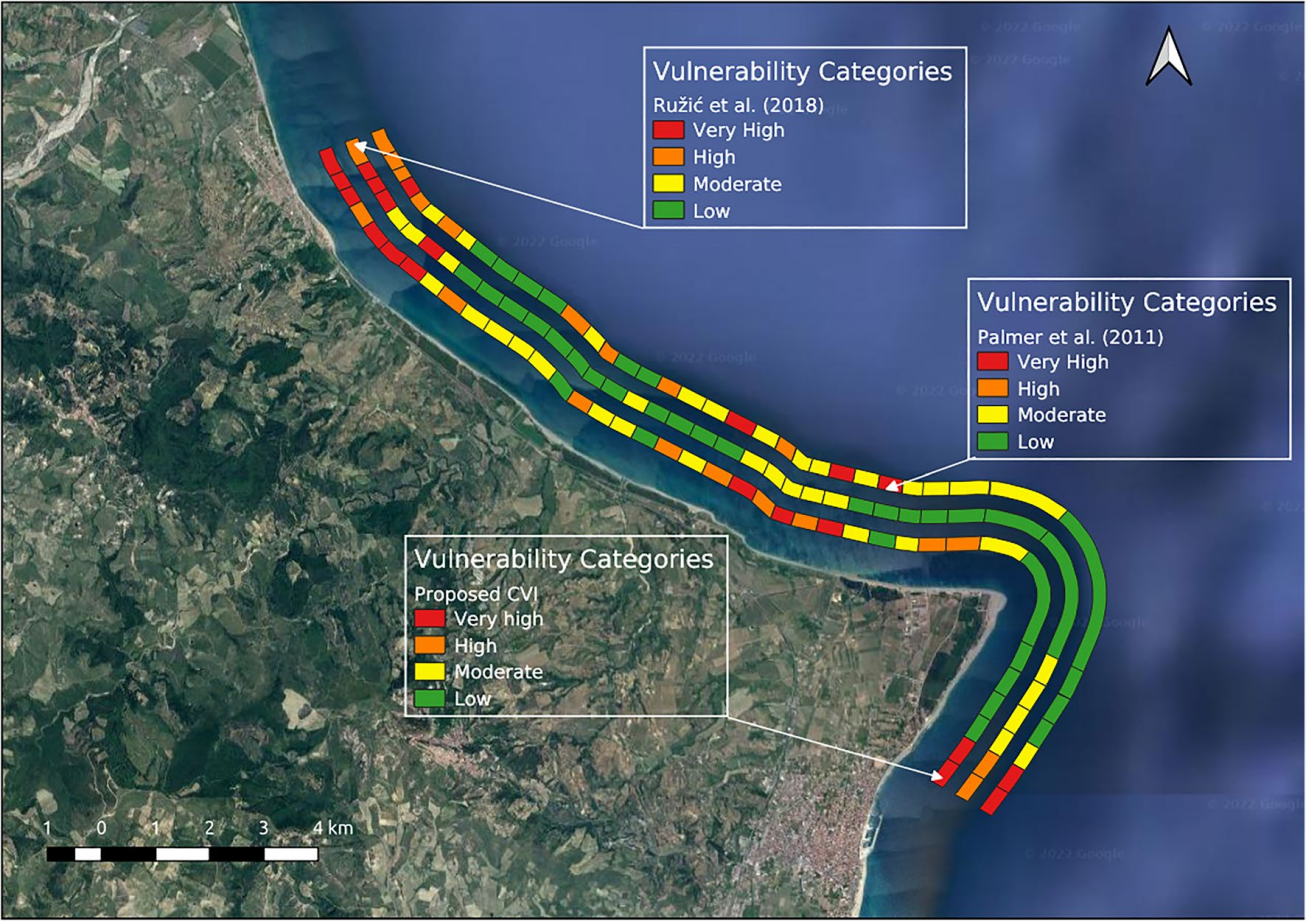
Proposed CVI formulation and CVI formulations proposed by Ružić et al.^46^ and Palmer et al.^47^. The map was created using QGIS 3.29 software (https://qgis.org).

Regarding the application and comparison of the proposed index with the previous formulation^27^, the results are shown in Fig. 6, while Fig. 7 shows the results of the comparison with the two other methods.

The results of the comparison of the proposed index with the previous authors’ CVI^27^ and the other two formulations^45,46^, are summarized in two tables included as Supplementary materials (Tables S.4, S.5).

## Discussion

Regarding the statistical analysis, the VIF values are within 10; therefore, this confirm that all the variables can be included in the index calculation. The results of the sensitivity analysis show that for all the considered variables, the correlation coefficients indicate a reduced sensitivity to variation in ranking score. The coverage of *Posidonia oceanica* has a lower correlation coefficient than the other variables, which indicates a greater sensitivity to changes. Considering the overall CVI output, the coefficient values are moderate with a greater sensitivity for the categorical scale, which uses equal classes. Regarding the sensitivity analysis of the aggregation method, the results of the two different mathematical formulations tested, show high correlation coefficients values. Similarly, the sensitivity analysis on the overall vulnerability categories rank denotes a low sensibility to change (r = 0,898).

Regarding the results of the CVI application, transects at low vulnerability are generally characterized by a wide or moderate emerged beach width and dune width. Dunes are generally of the hind dune type with a large or moderate width of vegetation behind the beach, mainly of the tertiary type. In particular, this category comprises 9 transects; transects 13, 14, and 18 are characterized by low vulnerability values mainly due to the presence of the dune system of “Dune of Marinella” consisting of width hind dunes and width secondary and tertiary vegetation, respectively. Transects 28, and 33–37 are at low vulnerability mainly due to the width of beaches and to the values of “Dune” and “Vegetation behind the back-beach” variables.

Transects at moderate vulnerability, similar to those at low vulnerability, are generally characterized by a wide or moderate emerged beach width. Dune width is generally moderate with hind dune and foredune types. The width of vegetation behind the beach is generally wide for all the transects, and the vegetation is of primary and secondary types. Compared to the previous category, these transects are characterized by lower coastal slopes and greater shoreline erosion conditions. Transects 8, 10–12, 16, 17 and 20 fall within the area of the “Dunes of Marinella” and therefore consist of areas with wide beaches and dunes; however these transects have a lower coastal slope and therefore a greater vulnerability value associated with this variable, a greater erosion condition and *Posidonia oceanica* is absent in some transects (16, 17). A similar condition concerns transects 27, 29 and 32 which are the last two transects of this category.

The transects at high vulnerability, are in areas with shoreline erosion conditions, the presence of small width dunes, primary or secondary vegetation and a low percentage of coverage of *Posidonia oceanica*, as in the case of transects 21, 23, and 25. This category also includes: transect 4, which is mainly characterized by a reduced beach width, without dunes or vegetation; transect 15, which has high vulnerability essentially due to the moderate beach width value and dune system damage; and transects 9 and 19, which are characterized by a small beach width, absence of streams and high erosion conditions. The last two transects of this category, 30 and 31, are mainly characterized by significant erosion conditions and the destruction of the dune system that in recent years, however, has begun to cover.

Finally, transects at very high vulnerability are characterized by high vulnerability values for almost all variables. In particular, for transects 1–3, and 5–7, the CVI values are very high due to the presence of an urbanized area with a reduced beach width, small distance of vegetation behind the beach, and absence of dune (or incipient and foredune type for transects 5 and 6); in the case of transects 22, 24 and 26, the CVI values are due mainly to the high erosion condition, by the presence of small width incipient dunes, by primary vegetation and by a low percentage of coverage of *Posidonia oceanica.* The last two transects of the category, 38 and 39, near the town of Cirò Marina, are at very high vulnerability, as these stretches are characterized by a moderate emerged beach width and a high vulnerability values for the remaining variables.

Regarding the comparison with the previous CVI formulation proposed by the authors, it should be noted that in transects 6, 22, 23 and 26, the CVI values increase in the present formulation, as all the added variables are characterized by high and very high vulnerability values.

Regarding transects 8, 15, 21 and 31, the vulnerability values increase compared to the previous formulation, varying from low to moderate vulnerability for transects 8 and 31 and from moderate to high vulnerability for transects 15 and 21. This increase in vulnerability is certainly due to *Posidonia oceanica* which has a high vulnerability value; even if the coverage of *Posidonia oceanica* results low, in the previous CVI formulation, the value associated with this variable would have been 1 (presence: very low). Instead, in the new formulation, the reduction in the defensive effects of the *Posidonia oceanica* is taken into account, since its coverage percentage is low. The river discharge variable also has high and very high values, which affects the increase in CVI value, while the other variables have moderate and low values.

Transects 9, 16 and 17 decrease the vulnerability value in the present formulation, varying from very high to high vulnerability (transect 9) and from very high to moderate (transects 16 and 17). For these transects, *Posidonia oceanica* had no influence, as it was not present. This variation in vulnerability is essentially due to the presence of hind dunes and primary and secondary vegetation which constitute an important barrier with respect to flooding for low-lying coasts.

Finally, for transects 18, 32, 34, 36 and 37 the decrease in vulnerability is due mainly to the values of the “Dune” and “Vegetation behind the back-beach” variables.

Overall, the comparison carried out showed that there are differences in vulnerability values due to the added variables. In particular, the dune, vegetation and coverage of *Posidonia oceanica* have a significant influences.

Regarding the method proposed by^45^, the geologic fabric variable assumes a constant value for all transects, similar to the geomorphology variable used in the index proposed in this paper, while the results relating to the coastal slope are different. In particular, considering the method proposed^45^, this variable assumes values between very low and low vulnerability (70% and 30% of the transects, respectively) while in the index proposed here, the values vary between high and very high vulnerability (51% and 49% respectively). This difference is due to the different ranking values and to the inverse score scale between the two indices. The beach width variable assumes constant values at low vulnerability considering the index proposed by^46^, while in the model proposed in this paper this variable assumes all the values from very low to very high vulnerability; this difference is due to the difference in the ranking values. The significant wave height variable assumes a constant value equal to high vulnerability while for land use the values vary from low to very high vulnerability (69%, 13%, 5%, 13% of transects, respectively). Regarding the overall CVI values obtained for each transect, it should be noted that this index shows a small variation in vulnerability classes. Approximately 49% of the transects are at low vulnerability, approximately 33.3% are at moderate vulnerability, 7.7% are at high vulnerability and 10% are at very high vulnerability. This small variation in vulnerability classes could make the index a weak tool for decision and management making processes for coastal areas, such as that of the case study, subject to different natural hazards and with limited resources to invest.

Regarding the index proposed by^46^, the beach width variable varies among all the vulnerability score classes, with a prevalence for the highest value of vulnerability (approximately 69% of transects). Similarly, the dune width variable assumes values between all vulnerability score classes but with a prevalence of transects at very low and low vulnerability (35,9% and 30,8% respectively). Regarding the distance to the 20 isobath variable, no transect has values at very low vulnerability while their prevalence assumes values of low vulnerability and high vulnerability in similar percentages. Regarding the width of the vegetation variable, the values vary among all vulnerability score classes but with a prevalence of transects at low vulnerability (approximately 56.4% of transects), while all transects have a percentage of outcrops corresponding to the highest value of the vulnerability score. Regarding the overall CVI values, approximately 25.6% of the transects are in the low vulnerability category, 35.9% are in the moderate vulnerability category, 23.1% are in the high vulnerability category and 15.4% are in the very high vulnerability category. Comparing the obtained results for the two methods, it should be noted that in the method proposed here, approximately 31% of the transects are at the same vulnerability category as that proposed by^46^, while approximately 46% of the transects show an increase in the vulnerability category when compared with that proposed by^46^. In particular, transects 1, 2 and 3 are in the very high vulnerability category in the present CVI formulation and in the high vulnerability category in the formulation proposed by^46^. This difference is due to the very high value of vulnerability score for all the other variables of the present CVI formulation. In transects 5, 6 and 7, all the variables except for two in the formulation proposed by^46^ have high vulnerability score values, while in the case of the present formulation, most of the variables have very high vulnerability score values leading to a greater vulnerability category in the present formulation. Similar considerations are for transects 16 and 17.

Regarding transects 9, 19, 22, 24, 25 and 30, while in both methods the common variables assume similar vulnerability score values, the increase in vulnerability category for the CVI proposed here is due to the high and very high values of all the other variables.

Regarding the transects characterized by a lower vulnerability in the present CVI index, it should be noted that in both methods the common variables assume similar vulnerability score values and the increase in vulnerability category considering Palmer’s method is due to the additional weighting of the intersects in an estuarine area, according to the mathematical formulation of the method, which is the case for transects 13, 18, 21, 28. Instead, for transects 14 and 37 the difference in vulnerability category is due to the low vulnerability score for some of the CVI variables in the present method.

Overall, the two methods are comparable even if the results of the application with the method proposed by^46^ show a lower number of transects in the very-high vulnerability category and a greater number in the moderate category; in the case, for example, of transects 6 and 22, characterized by high erosion conditions, low coastal slopes, no river/stream, low width of vegetation, and a very low percentage of *Posidonia oceanica* coverage, they variables result in a moderate vulnerability category, while with the index proposed by the authors, the transects are at very high vulnerability; this result appears more realistic for these transects. Overall, the use of variables such as dune and vegetation, coverage of *Posidonia oceanica*, coastal slope, and shoreline erosion accretion rates allow to obtain a more sensitive index with respect to inundation and extreme events; the index is therefore robust and useful for areas such as that of the case study, which are affected by different natural hazards and where planning and management actions must address the needs for economic development and the limitation of available financial resources to reduce threats and promote area development.

Finally, the obtained results have also been analysed considering the Regional Hydrogeological Plan of the Calabria Region (https://geoportale.regione.calabria.it); this plan uses a mathematical approach based on a set of data and parameters, to investigate different vulnerability threats that affect the regional territory such as flooding, coastal erosion, and hydraulic risk. It also includes programs, and structural and nonstructural interventions. In the Regional Hydrogeological Plan of the Calabria region areas between transects 1–7, 21–26, 38–39 are considered highly vulnerable to erosion, inundation and extreme events and are defined as areas that need more attention in planning and management activities. Considering the proposed CVI, transects 1–7, and 38–39, are at very high vulnerability (excepts transect 4), in agreement with the Regional Hydrogeological Plan, while the area between transects 21–26 has high and very high vulnerability. Therefore, the results of the proposed CVI formulation provide evidence similar to the results of the Regional Hydrogeological Plan and identify the areas that should be prioritized for interventions. It should be noted that a lack of other similar studies for the case study area did not allow for further comparisons and considerations.

It is worth noting that for transects 1–7, in the previous formulation proposed by the authors^27^, some transects are at high vulnerability; considering the index proposed by^45^, one transect is at high vulnerability, while two are at moderate vulnerability, while in the formulation proposed by^46^, almost the transects are at high vulnerability and one at moderate vulnerability. Therefore, the three formulations compared tend to consider the area least vulnerable. Regarding transects 21–26, the obtained results using the previous formulation^27^ are predominantly at moderate-high vulnerability; the method proposed by^45^ considers this area as a whole to be at low-moderate vulnerability, while Palmer's index considers it at higher vulnerability with transects at moderate, high and very-high vulnerability. Finally, considering the transect 38–39, in all formulations considered, the obtained results are similar (transects at very high vulnerability) even if Ružić’s formulation considers these transects to be at high vulnerability.

Therefore, the overall obtained results with the new CVI formulation find evidence with the real vulnerability of the study area and with the most vulnerable transects, and they better characterize the area than the other formulations.

## Conclusions

This paper proposes a coastal vulnerability index, in continuation with a previous work by the authors, suitable for low-lying Mediterranean coasts, and a case study has been presented. The main conclusions of the study are as follows.

The study area is characterized by low slopes and predominantly erosive conditions. The beaches of the study area are quite wide, and the majority of the area is characterized by a dune system comprising hind dunes, foredunes and incipient dunes; there are some stretches where the dune system has been damaged. The type of vegetation present is mainly tertiary vegetation: the width of the vegetation has mainly large values. The area is characterized by the presence of a few ditches and streams, and the coverage of *Posidonia oceanica* is low or even absent in various transects. The obtained results have shown that the most vulnerable areas are those near urban centers characterized by the absence of a dune system and vegetation behind the beach, while the low vulnerability transects are mainly characterized by wide/moderate emerged beach width, and the low vulnerability is also due to the characteristics of the dune system and vegetation. Sensitivity analysis showed that the index is lightly sensitive to changes in ranking scores, aggregation formulation and vulnerability classification method.

In coastal areas, such as that of the case study, characterized by low-lying coastlines, erosive conditions, and low slopes, affected by anthropogenic pressures, infrastructure, and growing tourist and economic activities, the proposed index could analyse the relative vulnerability of the different transects of the investigated coastal area, hence providing a reliable tool for local coastal management; this aspect is even more important to limited investment budgets. There is a need to define in detail the transects with the greatest vulnerability for which investments should be primarily focused. The lack of information and of systematic data collection on the Calabrian territory, prevented application to a longer stretch of coastline. Aware of this limitation, the aim of this work was to test the index, to assess its feasibility and initially apply it to identify the vulnerable coastal areas that should be prioritized. Future research objectives include the application of the proposed index to a larger spatial scale with greater variable heterogeneity.

Regarding the comparison with the previous formulation proposed by the authors the variables “Dune” and “Vegetation” has provided more information on the ability of the method to counteract the action of floods/storm surges and the dissipation of wave energy; in addition, for an area characterized by shoreline erosion and changes in land use, the integration of the variable “River discharge” allowed us to account for the effects of rivers to limit erosive conditions. The redefinition of *Posidonia oceanica* also had a significant influence on the final results of the application, as it allowed for a more detailed analysis compared to only estimating its presence/absence, asits defence action varies in relation to its coverage degree. A comparison of the results showed that the contribution of these variables, completes the previous formulation, and the application showed a difference in the vulnerability categories with a greater number of transects at very high vulnerability and moderate vulnerability and a reduction in those at low vulnerability. These results find evidences on the real vulnerability of the territory.

The comparison with the other two methods also indicated that the results of the proposed index appear more realistic for the case study area and that the set of variables used provides a robust analysis of the different threats and defence/protection factors that influence the overall vulnerability value for the typology of coastal area studied. To find evidence on the real vulnerability of the territory, a regional study has also been considered; the lack of other studies on this issue in the case study area did not permit further comparisons. Overall, the results of this first application appear consistent with the study area conditions, and the aim of future research is to validate the proposed index by comparing it with more complex numerical models to make the index a useful tool for coastal planning and management.

## Supplementary Information

Supplementary Information.

## Data Availability

The dataset used and/or analysed during the current study is available from the corresponding author on reasonable request.
