# Supplementary material for: Improvement of a coastal vulnerability index and its application along the Calabria Coastline, Italy
Source: Sci Rep. 2022 Dec 19;12:21959. doi: 10.1038/s41598-022-26374-w (PMC9763354; doi:10.1038/s41598-022-26374-w)
Supplement: Supplementary file 1 — Supplementary Information. [file 41598_2022_26374_MOESM1_ESM.pdf]

Improvement of a coastal vulnerability index and its application along the Calabria Coastline, Italy.

Daniela Pantusa, Felice D'Alessandro, Ferdinando Frega, Antonio Francone and Giuseppe Roberto Tomasicchio

Table S.1. Variables and ranking values for CVI methods proposed by Pantusa et al., (2018)

METHOD	VARIABLES	SCORE				
		**Very low (1)**	**Low (2)**	**Moderate (3)**	**High (4)**	**Very high (5)**
Pantusa et al. (2018)	Geomorphology	Rocky, cliffed coasts	Medium cliffs, indented coasts	Low cliffs, alluvial plains	Cobble beaches, estuary, lagoon	Barrier beaches, sand beaches, salt marsh, mud flats, deltas, coral reefs
	Coastal slope (%)	>12	8–12	4–8	2–4	<2
	Shoreline Erosion/accretion (m/year)	>(+ 1.5)	(+1.5)–(+0.5)	(−0.5)–(+0.5)	(−0.5)–(−1.5)	<(−1.5)
	Emerged beach width (m)	>100	50–100	25–50	10–25	<10
	Dune width (m)	>100	75–100	50–75	25–50	<25
	Relative sea-level change (mm/year)	<1.8	1.8–2.5	2.5–3.0	3.0–3.4	>3.4
	Mean significant wave height (m)	<0.3	0.3–0.6	0.6–0.9	0.9–1.2	>1.2
	Mean tide range (m)	>0.8	0.6–0.8	0.4–0.6	0.2–0.4	<0.2
	Width of vegetation behind the beach (m)	>400	200–400	100–200	50–100	<50
	Posidonia oceanica (Boolean: presence/absence)	Present				Absent

Table S.2 Variables and ranking values
CVI methods proposed by Palmer et al., (2011) and Ružić et al. (2019)

**Method**	**Variables**	**Score**				
		**Very low (1)**	**Low (2)**	**Moderate (3)**	**High (4)**	**Very-high (5)**
Palmer et al. (2011)	**Parameter**	**Score**				
		**Extremely low (1)**		**Low (2)**	**Moderate (3)**	**High (4)**
	Beach width (m)	> 150		100-150	50-100	< 50
	Dune width (m)	> 150		50-150	25-50	< 25
	Distance to 20m isobath (Km)	> 4		2-4	1-2	< 1
	Distance of vegetation behind the back beach (m)	>600		200-600	100-200	< 100
	Percentage outcrop (%)	>50		20-50	10-20	<10
Ružić et al. (2019)	**Parameter**	**Score**				
		**Very low (1)**	**Low (2)**	**Moderate (3)**	**High (4)**	**Very-high (5)**
	Geologic fabric	Proluvial sediments	Resistant carbonate rocks	Crushed carbonate rocks	Talus breccia	Flysch rocks and colluvium
	Coastal slope (º)	<5	5-12	12-20	20-32	>32
	Beach width (m)	> 15	10-15	5-10	2,5-5	<2.5
	Significant wave height Hs (m)	< 0,35	0,35-0,70	0,70-1,05	1,05-1,4	>1,40
	Land Use	Bare karst	Mediterranean vegetation cover	Beach	Touristic zone	Settlement

Table S.3 Vulnerability value for each variable and CVI values for each transect

Transect	Geomorphology	Costal Slope	Shoreline erosion/ accretion rate	Emerged Beach Width	Dune	River discharge	Rate of relative sea-level rise	Mean significant wave height	Mean tide range	Vegetation behind the back-beach	Coverage of *Posidonia oceanica*	CVI value	CVI category
1	5	5	2	3	5	5	5	4	5	5	4	825.72	Very high
2	5	4	5	3	5	5	5	4	5	5	3	1011.30	Very high
3	5	4	2	3	5	5	5	4	5	5	4	738.55	Very high
4	5	4	2	2	5	4	5	4	5	5	4	539.36	High
5	5	4	5	3	3	5	5	4	5	4	4	809.04	Very high
6	5	4	4	3	3	5	5	4	5	4	4	723.63	Very high
7	5	4	4	3	5	5	5	4	5	3	4	809.04	Very high
8	5	4	4	3	2	4	5	4	5	2	4	373.68	Moderate
9	5	5	5	3	1	5	5	4	5	2	5	412.86	High
10	5	5	5	3	1	5	5	4	5	2	4	369.27	Moderate
11	5	5	4	3	1	5	5	4	5	2	4	330.29	Moderate
12	5	4	5	3	1	5	5	4	5	2	4	330.29	Moderate
13	5	5	5	3	2	3	5	4	5	2	4	404.52	Moderate
14	5	5	1	4	2	5	5	4	5	2	4	269.68	Low
15	5	4	5	3	2	4	5	4	5	2	4	417.79	High
16	5	4	5	3	1	5	5	4	5	2	5	369.27	Moderate
17	5	4	5	3	1	5	5	4	5	2	5	369.27	Moderate
18	5	4	5	3	1	4	5	4	5	2	4	295.42	Low
19	5	5	5	4	1	5	5	4	5	2	4	426.40	High
20	5	5	4	3	1	5	5	4	5	2	4	330.29	Moderate
21	5	5	4	3	2	4	5	4	5	2	4	417.79	High
22	5	4	5	3	4	5	5	4	5	3	4	809.04	Very high
23	5	4	2	4	4	5	5	4	5	2	4	482.42	High
24	5	5	4	3	4	5	5	4	5	2	4	660.58	Very high
25	5	5	4	2	4	5	5	4	5	2	4	539.36	High
26	5	5	4	3	4	5	5	4	5	2	4	660.58	Very high
27	5	5	4	3	2	5	5	4	5	1	4	330.29	Moderate
28	5	5	4	3	1	4	5	4	5	1	4	208.89	Low
29	5	5	4	3	1	5	5	4	5	2	4	330.29	Moderate
30	5	5	5	2	3	5	5	4	5	3	4	639.60	High
31	5	5	5	2	2	5	5	4	5	2	4	426.40	High
32	5	5	5	2	2	5	5	4	5	1	5	337.10	Moderate
33	5	5	1	1	1	5	5	4	5	1	5	75.38	Low
34	5	4	4	2	1	5	5	4	5	1	5	190.69	Low
35	5	4	4	2	1	5	5	4	5	1	4	170.56	Low
36	5	4	4	2	1	5	5	4	5	1	5	190.69	Low
37	5	4	4	2	1	5	5	4	5	2	5	269.68	Low
38	5	4	4	2	5	5	5	4	5	5	4	852.80	Very high
39	5	4	4	3	5	5	5	4	5	5	5	1167.75	Very high

Table S.4. Comparison of the proposed CVI and the previous CVI formulation developed by the authors (Pantusa et al., 2018)

Transects	Proposed CVI formulation	Previous CVI formulation
1	Very high	Very high
2	Very high	Very high
3	Very high	Very high
4	High	High
5	Very high	Very high
6	Very high	High
7	Very high	Very high
8	Moderate	Low
9	High	Very high
10	Moderate	Moderate
11	Moderate	Moderate
12	Moderate	Moderate
13	Moderate	Moderate
14	Low	Low
15	High	Moderate
16	Moderate	Very high
17	Moderate	Very high
18	Low	Moderate
19	High	High
20	Moderate	Moderate
21	High	Moderate
22	Very high	High
23	High	Moderate
24	Very high	High
25	High	High
26	Very high	High
27	Moderate	Moderate
28	Low	Low
29	Moderate	Moderate
30	High	High
31	High	Low
32	Moderate	High
33	Low	Low
34	Low	Moderate
35	Low	Low
36	Low	Moderate
37	Low	High
38	Very high	Very high
39	Very high	Very high

Table S.5. Comparison of the proposed CVI and the CVI formulations proposed by Ružić et al. (2019) and Palmer et al. (2011)

Transects	Proposed CVI formulation	CVI formulation proposed by Ružić et al. (2019)	CVI formulation proposed by Palmer et al. (2011)
1	Very high	High	High
2	Very high	Very high	High
3	Very high	Very high	High
4	High	Very high	Very high
5	Very high	Moderate	High
6	Very high	Moderate	Moderate
7	Very high	Very high	High
8	Moderate	Moderate	Moderate
9	High	Low	Low
10	Moderate	Low	Low
11	Moderate	Low	Low
12	Moderate	Low	Low
13	Moderate	Low	High
14	Low	Low	Moderate
15	High	Low	High
16	Moderate	Low	Low
17	Moderate	Moderate	Low
18	Low	Low	High
19	High	Low	Moderate
20	Moderate	Low	Moderate
21	High	Low	Very high
22	Very high	Moderate	Moderate
23	High	Moderate	High
24	Very high	Moderate	Moderate
25	High	Moderate	Moderate
26	Very high	Moderate	Very high
27	Moderate	Low	Moderate
28	Low	Low	Very high
29	Moderate	Low	Moderate
30	High	Low	Moderate
31	High	Low	Moderate
32	Moderate	Low	Moderate
33	Low	Low	Low
34	Low	Moderate	Low
35	Low	Moderate	Low
36	Low	Moderate	Low
37	Low	Moderate	Moderate
38	Very high	High	Very high
39	Very high	High	Very high
